# South Africa’s COVID-19 Alcohol Sales Ban: The Potential for Better Policy-Making

**DOI:** 10.34172/ijhpm.2020.93

**Published:** 2020-06-10

**Authors:** Richard Matzopoulos, Helen Walls, Sarah Cook, Leslie London

**Affiliations:** ^1^School of Public Health & Family Medicine, University of Cape Town, Cape Town, South Africa.; ^2^Burden of Disease Research Unit, South African Medical Research Council, Cape Town, South Africa.; ^3^Faculty of Public Health and Policy, London School of Hygiene and Tropical Medicine, London, UK.; ^4^Faculty of Epidemiology and Population Health, London School of Hygiene and Tropical Medicine, London, UK.; ^5^Department of Community Medicine, UiT the Arctic University of Norway, Tromsø, Norway.

## Dear Editor,

Evidence of the social impacts of emergency responses to coronavirus disease 2019 (COVID-19) is emerging in several countries, including increased prevalence of domestic violence.^1^ Alcohol is the elephant in the room in mediating many such impacts, yet governments have been reluctant to address alcohol sales in the context of social-distancing measures. Exceptions include several African countries including South Africa, Namibia, and Madagascar.^2^ The dramatic decrease in violence and injuries following an alcohol sales ban in South Africa has implications for its alcohol policy post-lockdown as well as the current emergency response in other countries.

In mid-March 2020 South Africa limited alcohol sales after 6 pm and restricted establishments selling alcohol to a maximum of 50 patrons. Subsequently, a national lockdown prohibited the sale of all liquor. These measures are considerably stricter than those of other countries. In the United Kingdom and New Zealand, for example, allowances have been made for alcohol purchases ostensibly out of concern for sudden/unsupported withdrawal on dependent people.

The rationale in South Africa for implementing stricter measures is founded partly on alcohol’s role in undermining social distancing and compromising immune response. However, the government’s resolve seems strengthened by alcohol’s contribution to the high levels of interpersonal violence and injuries. Violence-related trauma admissions and homicides in South Africa are strongly associated with drinking. Men constitute the overwhelming majority of victims and are also the main perpetrators of interpersonal violence.^3^ With men confined to their homes during lockdown, increased domestic violence is a concern.

These dynamics appear to have been confirmed by both early reports of a significant reduction in trauma unit admissions for alcohol-related injury^4^ and a reported increase in domestic violence complaints to the police.^5^ The decline in alcohol-related trauma, alongside alcohol’s perceived role in crime and undermining public safety, has won political and popular support for maintenance of these restrictions.^6^ The national police minister has even seemingly expressed a desire for a permanent prohibition,^7^ although historical precedents and the enduring impasse of the war on drugs suggest that prohibitions should only ever be considered as temporary measures. More importantly, the South African public’s recognition that controlling alcohol availability during the lockdown was in the interest of the public good suggests that the sales ban was akin to a public health “teachable moment” – the impact of alcohol has become immediately apparent through its absence. This should resonate in settings where alcohol fuels violence and injuries and plays a similar role in undermining the COVID-19 response. It also has future relevance given that lockdowns of various forms and intensity are likely to continue in many countries for the foreseeable future.

South Africa’s rapid and forceful restrictions on alcohol under COVID-19 are in stark contrast to previous resistance to policy changes to address the main drivers of alcohol harms at the population level. Alcohol imposes an enormous toll on South African society through associated economic, social and health costs – estimated at 10%-12% of gross domestic product.^8^ Despite this, lobbying efforts by South Africa’s powerful alcohol industry have ensured that business interests take precedence.^9^ The Control of Marketing of Alcoholic Beverages Bill placed before Cabinet in 2013 restricts advertising/marketing of alcoholic beverages except at point of sale, sponsorship and promotion of alcoholic beverages. The Bill has been subjected to two regulatory-impact and one socio-economic assessment, none of which have been made public. Two other policy documents, the National Draft Liquor Bill and Western Cape Alcohol Harms Reduction Policy, are both currently stalled. Meanwhile alcohol has become 60% more affordable in South Africa over the past 50 years.^10^

While the Sustainable Development Goals recognise alcohol as a developmental impediment, any new approach to regulation in South Africa needs to consider wider impacts on the ‘alcohol environment,’ including health and social outcomes. Measures taken because of COVID-19 – and later counter strategies – all shape this dynamic environment.^11^ If restrictions on the alcohol trade are to be considered post-lockdown they must be designed not only to reduce harm, but also to mitigate against unintended consequences. For example, approximately 50 000 South Africans derive subsistence livelihoods from the informal alcohol trade.^12^ While there may now be impetus for tighter restrictions on the retail environment, these should be accompanied by investment in alternative income opportunities. Industry-aligned groups have also highlighted the plight of alcoholics suffering unplanned withdrawals during lockdown. Therefore management of alcohol dependency needs to be included explicitly as part of the overarching strategy to reduce alcohol harm.

In the short-term the current alcohol sales ban will need careful phase-out to prevent a subsequent surge in drinking. The end-point should at least ensure that national norms and standards for retailer operating hours devised in 2015 are included in the National Liquor Act and applied throughout the country. Policy advocates and activists must not only use the current moment as a political ‘window of opportunity,’ but must also use it to highlight the enduring nature of the alcohol – trauma nexus. Reliable, current data are essential to show the association between alcohol availability and injury caseloads clearly and to ensure that the appetite for public health-oriented policy extends beyond the current crisis. This applies equally to countries considering the implementation of similar restrictions.

Much is being discussed currently about how countries can ease lockdown and get ‘back to normal.’ In South Africa, where the alcohol ban differs hugely from the easing of social-distancing measures is that returning alcohol availability to ‘normal’ will return us to situations of highly hazardous use. Instead, we have here an opportunity for stakeholders to work together to develop better alcohol policy and safeguard the post-COVID future of all South Africans. The country’s response will set an important precedent for countries elsewhere confronting similar challenges.

## Ethical issues

Not applicable.

## Competing interests

Authors declare that they have no competing interests.

## Authors’ contributions

RM, HW, SC, LL were responsible for the conception and design of the manuscript. RM drafted the manuscript. RM, HW, SC, and LL critically revised and made important intellectual contributions to the manuscript.

## Funding

RM is funded by the South African Medical Research Council.

## Authors’ affiliations

^1^School of Public Health & Family Medicine, University of Cape Town, Cape Town, South Africa. ^2^Burden of Disease Research Unit, South African Medical Research Council, Cape Town, South Africa. ^3^Faculty of Public Health and Policy, London School of Hygiene and Tropical Medicine, London, UK. ^4^Faculty of Epidemiology and Population Health, London School of Hygiene and Tropical Medicine, London, UK. ^5^Department of Community Medicine, UiT the Arctic University of Norway, Tromsø, Norway.
